# Unlocking the potential of social prescribing for healthy ageing in the Western Pacific

**DOI:** 10.1016/j.lanwpc.2025.101760

**Published:** 2025-12-15

**Authors:** Siwon Lee, Mikiko Kanda, Hiromasa Okayasu

**Affiliations:** World Health Organization (WHO) Regional Office for the Western Pacific, Manila, Philippines

The recent report by the WHO Commission on Social Connection highlighted the urgent need to address loneliness and social isolation, which particularly affects older adults.^1^ Social disconnection directly influences biological, psychological, and behavioural pathways to health, reinforcing the role of social connection as a foundation for health and healthy ageing.^1^ Social prescribing provides a practical approach to strengthening these connections by linking individuals to community activities and services that foster social participation.^2^

In Europe, only 10% of the self-reported health gap between the most and least affluent adults is due to differences in healthcare access and quality.^3^ The remaining 90% stems from non-health factors—such as income security, housing, education, social protection, and working conditions.^3^ This highlights the need to address broader social determinants of health to ensure more equitable health outcomes. Due to life–stage transitions, greater attention should be given to socio-economic factors, digital literacy, social cohesion, social isolation, and transportation challenges affecting older people's health.^4^ In Japan, low socio-economic status poses a substantial health risk for older adults.^5^ Older men with lower education or income levels were found to have a 1.6–2.0 times greater risk of premature death.^5^

Emerging evidence from the Western Pacific suggests social determinants of health have similar impact on health outcomes of older individuals. This need to address broad social determinants of health is especially urgent in the WHO Western Pacific Region, home to one of the largest and fastest-growing older populations globally.^2^ A key pillar of healthy ageing is social well-being, making social isolation a critical public health concern. In the Western Pacific, the prevalence of loneliness is estimated at 11%, and globally, 25–34% of older adults experiences social isolation.^1^ These conditions are associated with a 9–33% higher risk of mortality, comparable to major risk factors like smoking, physical inactivity, or air pollution.^1^ Social isolation also contributes to 5% of global dementia cases—especially concerning for older adults.^6^ Isolated older Japanese individuals had 1.3–1.8 times the risk of functional disability.^5^

Social prescribing offers a promising approach to address social isolation, loneliness, and broader determinants of health. It enables healthcare professionals to refer patients to non-medical services—such as arts and cultural activities, and social welfare services—that address social needs and enhance overall well-being.^7^ By connecting individuals with community-based resources, social prescribing helps build social connections and supports functional ability, promoting greater independence, and improved quality of life. Emerging evidence from the United Kingdom suggests that social prescribing can reduce healthcare utilization, including emergency department visits and hospital admissions, with particularly significant benefits for older adults.^7^^,^^8^ Despite the potential impact of social prescribing on health, social prescribing research in the Western Pacific region is still in preliminary stage.

The WHO Regional Office for the Western Pacific has been promoting social prescribing since 2021 as part of broader efforts to strengthen collaboration between the health and non-health sectors, advocating for better conditions outside the health sector for improved health outcomes. To unlock the full potential of social prescribing in the Western Pacific, governments, healthcare providers, and community organizations must invest in research and policy development. Depending on the context, social prescribing implementation models vary. In the UK, the National Health Service England employs link workers to be based in primary care clinics to help patients access community resources aligned with their interests and needs.^9^ In Singapore, SingHealth Community Hospitals’ link workers support patients transition from hospital to home by ensuring they have appropriate support in their community.^10^ Culturally sensitive research is critical to refining models that meet the diverse needs of different populations. Embedding social prescribing into existing health systems—including primary health care, long-term care, and age-friendly initiatives—is essential for long-term success in supporting healthy ageing.

Academia is actively engaged in building the evidence base for the benefits of social prescribing. The social prescribing in the Western Pacific region Series provides a platform for showcasing regional experiences and informing policy development. It highlights research to date from our region and encourages further evidence-building from the Western Pacific. This collection also highlights models that can be adapted for low-resource settings and help drive evidence-informed implementation across the region.

Healthy ageing requires a holistic approach that addresses not only physical and mental health, but also social well-being.^2^ By embedding social prescribing into age-friendly policies, health systems, and social protection frameworks, countries can take a life course approach to healthy ageing–adding life to years. Continued research, cross-sector collaboration, and cultural adaptation in social prescribing will support healthy ageing in the region.

## Declaration of interests

The authors declare that they have no competing interests.
